# The contribution of LepA to virulence and host cell invasion in *Salmonella* Typhimurium

**DOI:** 10.1186/s12866-026-05826-7

**Published:** 2026-10-22

**Authors:** Sebastian Braetz, Andreas Nerlich, Marcus Fulde

**Affiliations:** 1 Institute of Microbiology and Epizootics, School of Veterinary Medicine, Freie Universität Berlin, Berlin, Germany; 2 Veterinary Centre for Resistance Research (TZR), School of Veterinary Medicine, Freie Universität Berlin, Berlin, Germany; 3 Present Address: Institute of Microbiology, University of Veterinary Medicine Hannover, Bischofsholer Damm 15, 30173 Hannover, Germany

**Keywords:** *Salmonella*, LepA, bacterial invasion, virulence regulation, host-pathogen interaction, *Galleria mellonella* infection model

## Abstract

**Background:**

LepA is one of the most conserved proteins and is found in nearly all bacteria as well as in eukaryotes. Mutation of *lepA* increases antibiotic tolerance in *Escherichia coli*, *Salmonella*, and rifampicin-resistant clinical mycobacterial isolates. However, the physiological function and its role in virulence of LepA remain elusive, particularly considering that LepA is non-essential under laboratory conditions.

**Methods:**

We investigated the *lepA* gene of *Salmonella* Typhimurium for its role in bacterial invasion of host cells. Invasion assays using *lepA* mutants were performed in macrophages (J774) and epithelial cells (Caco-2 and HT29-MTX), both in the presence and absence of mucus. In addition, virulence gene induction, motility, and pathogenicity in a *Galleria mellonella* infection model were assessed.

**Results:**

The *lepA* deletion strain showed reduced invasion in all cell types regardless of mucus, indicating its essential role in the invasion process and in the initial survival within macrophages. Deletion of *lepA* also resulted in decreased induction of virulence-associated genes and reduced pathogenicity in *Galleria mellonella*.

**Conclusion:**

These findings provide important insights into the molecular determinants of *Salmonella* Typhimurium virulence and contribute to a deeper understanding of the conservation of LepA across organisms.

**Supplementary Information:**

The online version contains supplementary material available at https://doi.org/10.1186/s12866-026-05826-7.

## Introduction

*Salmonella* is a rod-shaped, facultative intracellular pathogen comprising more than 2500 serovars, with *Salmonella enterica* subspecies *enterica* being the most clinically relevant pathogenic subspecies [1]. These subspecies cause 99% of the infections in humans and animals, leading to gastroenteritis, enteric fever, bacteremia, and a chronic carrier state. Among the *enterica* subspecies, Typhimurium is the most prevalent serovar, responsible for approximately 12% of infections [1, 2]. Contamination can occur via uncooked poultry, fruits, vegetables, as well as eggs and dairy products [3–5]). Consumption of contaminated food can cause diverse symptoms, including nausea, diarrhea, vomiting, and fever. For elderly people, children, and immunocompromised patients, such infections can become life-threatening and may require antibiotic therapy, which in turn increases the risk of recalcitrant and recurring infections due to antibiotic-tolerant persister cells that can further develop antibiotic resistance [1, 6, 7]. The colonization of the gut and invasion into the epithelial cells of the host is a strictly regulated system, involving diverse activators and repressors to allow expression of the *Salmonella* pathogenicity island 1 (SPI-1) at the right moment [8]. SPI-1 encodes a type III secretion system (T3SS), a needle-like structure on the cell surface of *Salmonella* that injects effector proteins into the host cells to stimulate the invasion and uptake of *Salmonella* by remodeling the cytoskeleton of the host cells. In addition, SPI-1 modulates the immune response and inhibits a cytokine-mediated pro-inflammatory reaction by immune cells [9]. The major activator of SPI-1 is HilA, which is regulated by several proteins [8, 10]. The inhibition and derepression of HilA strongly depend on environmental factors such as changes in osmolarity, pH, and oxygen tension, as well as the presence of bile acids, short-chain fatty acids, and glucose availability sensed by *Salmonella* [8, 11]. After internalization into mammalian cells, Salmonella pathogenicity island 2 (SPI-2), which is another T3SS, is activated [12]. It translocates various effector proteins into the cytosol of the host cell, facilitating the establishment and maturation of the *Salmonella*-containing vacuole (SCV) for intracellular proliferation. Furthermore, SPI-2 interferes with the fusion of lysosomes after internalization into macrophages, protecting *Salmonella* from enzymatic degradation and killing [13]. This protective mechanism allows *Salmonella* to proliferate within macrophages, increasing the risk of systemic infection.

LepA (elongation factor 4) has not yet been associated with the virulence of *Salmonella* or other pathogens. So far, it has been reported that the disruption of *lepA* in *Helicobacter pylori* decreases the bacterium’s capability to grow under low pH conditions [14], which may reduce its pathogenicity during colonization of the stomach. Furthermore, mutations within *lepA* in rifampicin-resistant *Mycobacterium tuberculosis* isolates were reported to cause *rpoB* overexpression [15]. Deletion of *lepA* in *E. coli* increased drug tolerance to various classes of antibacterial antibiotics due to reduced reactive oxygen species (ROS) formation and MazEF-mediated cell death [16]. In *Salmonella* Typhimurium, deletion of *lepA* resulted in increased persister cell survival following ciprofloxacin treatment due to reduced ROS-dependent activation of prophages and reduced prophage-mediated killing [17]. These studies indicate that LepA may also have an important role from a clinical perspective. Some studies propose that LepA is involved in translational proofreading [18, 19], while others claim that it plays a role in the initiation of translation, contributes to the biogenesis of ribosomes, or reactivates stalled ribosomes on mRNA at high magnesium concentrations or low temperature [20–22]. Interestingly, LepA is one of the most conserved proteins and can be found in almost all bacterial species, as well as in mitochondria and chloroplasts. However, under optimal growth conditions, LepA is dispensable for proliferation [19, 23, 24]. Therefore, we were interested in studying the physiological role of LepA in *Salmonella* and how this highly conserved protein influences bacterial physiology and contributes to the organism’s pathogenicity. In this study, we highlight the role of LepA in the efficient invasion of eukaryotic host cells and its impact on bacterial survival in hostile environments during infection in *Galleria mellonella* larvae, providing new insights into the infection mechanisms of *Salmonella* and, in turn, helping to explain the conservation of LepA across organisms.

## Materials and methods

### Bacterial and eukaryotic strains with their growth conditions

The bacterial strains and eukaryotic cells are listed in Table S1 (supplementary data). The wild type strain is *Salmonella enterica* serovar Typhimurium (ATCC 14028) ([25]; Braetz et al., 2022). All mutants are based on this wild type strain. To knockout the respective genes, we conducted λ Red recombinase mutagenesis as described by Datsekno and Wanner [26]. Primers used in this study are summarized in Table S3. After electroporation, the bacteria were plated on LB containing 50 µg/ml kanamycin for selection. Successful deletion was confirmed by PCR. To eliminate the kanamycin-resistant cassette, the mutant strains were electroporated with pCP20 and incubated at 29 °C. For the removal of pCP20, the bacteria were shifted to growth at 37 °C. Eventually, all mutants were tested for loss of kanamycin and ampicillin resistance.

To construct the SPI-1 and SPI-2 reporters, we used the *PprgH*-*gfp* (SPI-1) and *PssaG*-*gfp* (SPI-2) constructs inserted into the *putPA* locus. For this purpose, we obtained the strain JH3010 (SPI-1)/ JH3009 (SPI-2) from Hautefort et al., [27] and generated a P22 phage lysate from these strains. Using these lysates, we transduced the *PprgH-gfp*/*PssaG-gfp* fusion into our own strains. To avoid carryover of P22-infected transductants, we purified putative mutants on green plates [28].

### Invasion assays

For invasion assays, Caco-2 cells and J774A.1 macrophages (both obtained from ATCC) were seeded in 48-well plates and incubated at 37 °C with 5% CO₂ until confluency was reached. Caco-2 cells were grown in DMEM supplemented with 10% fetal bovine serum (FBS), while J774 macrophages were grown in Iscove modified Dulbecco’s medium (IMDM) with FBS. The bacteria were incubated in Lennox Broth medium (LB) to an OD_600_ of 2.0–2.5. After reaching the appropriate cell density, the bacteria were centrifuged and resuspended in the respective cell medium. After preparing the bacteria, the host cells were washed twice with 1x PBS, and 1 ml of culture medium containing the bacteria at a multiplicity of infection (MOI) of 10 was added to the host cells in duplicate for each strain. The plate was then centrifuged for 10 min at 250 x g. For the invasion assays with Caco-2 cells, bacteria and host cells were incubated for 60 min at 37 °C and 5% CO_2_, while for invasion into J774 macrophages, only 30 min were used. Subsequently, the infected wells were washed twice with PBS, and cell culture medium containing 50 µg/ml gentamicin was added to kill the remaining non-invasive bacteria for 30 min. As an internal control, we plated aliquots (10 µL) of the supernatant on LB agar plates to ensure that all bacteria had been killed by gentamicin. Following gentamicin treatment to kill non-invasive bacteria, host cells were washed twice with 1x PBS and subsequently lysed with Triton X-100 (0.1%). The invasion rate is presented as colony-forming units per well in the figures. Invasion assays following incubation to the post-exponential phase (OD_600_ = 1.0) were performed as described above.

Invasion assays into HT29-MTX cells (obtained from Merck) were conducted similarly with some modifications. HT29-MTX cells were seeded into 48-well plates and incubated in DMEM + FBS + non-essential amino acids (1:10) until confluency. For mucus formation, HT29-MTX cells were incubated for 21 days. The thickness of the mucus layer was determined under the microscope using glass beads, indicating that the mucus produced by HT29-MTX cells is approximately 25 μm thick. To avoid damaging the mucus layer produced by the epithelial cells, the plate was not centrifuged as in the previous assays. Instead, 200 µl of the bacterial suspension was carefully added to the cell surface and incubated at 37 °C and 5% CO_2_ for 60 min. Subsequently, the procedure was as described above.

### Adhesion Assay

Determination of adhesion to the cell surface of Caco-2 cells was conducted according to Chessa et al., [29]. Prior to the adherence assay, Caco-2 cells were washed with cold PBS and incubated in culture medium for one hour at 4 °C. In parallel, the bacteria were prepared as for the invasion assays and diluted in culture medium (DMEM + 10% FBS) before applying a thin layer of bacterial suspension onto the host cells. Subsequently, the plates were further incubated for one hour at 4 °C. The low temperature prevents invasion and allows quantification of bacteria attached to the Caco-2 cell surface. After incubation, each well was washed four times with PBS, and the Caco-2 cells were lysed with 0.1% Triton X-100 for CFU determination. As a control, after the washing steps, culture medium containing 50 µg/ml gentamicin was added to designated wells to kill extracellular bacteria. The plates were incubated for 30 min at 37 °C, subsequently washed, and lysed as described above for CFU determination. This procedure allows differentiation between adherent (non-invasive) and invasive bacteria.

### LacZ-Assay

The bacteria were incubated as described above for the invasion assays in LB to determine β-galactosidase activity controlled by the promoters of *hilA* and *sipC* [11]. Subsequently, 1 ml of culture was removed and centrifuged for 10 min at 6,000 x g at 4 °C. The pellet was resuspended in Z-buffer (60 mM Na₂HPO₄, 40 mM NaH₂PO₄, 10 mM KCl, 1 mM MgSO₄, 50 mM β-mercaptoethanol, pH 7.0) at a 1:10 dilution, and the optical density at 600 nm (OD₆₀₀) was measured. The suspension was equilibrated at 28 °C, and 200 µl ONPG (o‑Nitrophenyl-β-D-galactopyranosid, 4 mg/ml) was added, followed by incubation at 28 °C. The time until the solution turned yellow was recorded. The reaction was stopped with Na₂CO₃, 1 ml of the sample was removed, and the mixture was centrifuged for 5 min at 16,000 x g. The supernatant was measured at OD₄₂₀ and OD₅₅₀ to calculate Miller Units, normalized to the OD₆₀₀ of the culture. As a control, the wild type (8640) without the *lacZ* gene was used to subtract the background signal.

### FACS analysis

To determine the induction of P*prgH*-*gfp* under various conditions, bacteria were incubated in LB medium at 37 °C with shaking until reaching an OD_600_ of 2.0–2.5 or OD_600_ = 1.0. Following incubation, the cells were washed with PBS and resuspended in one of the following media: M9 + 0.4% glucose, M9 + 4% glucose, M9 + 0.4% glucose without MgSO₄, M9 + 5 mM acetate, DMEM + 10% FCS, or LB. The suspensions were then incubated for one hour at 37 °C. For low oxygen (microaerophilic) conditions, bacteria resuspended in M9 + 0.4% glucose or in DMEM were placed in a cell culture incubator with 5% CO₂. The samples resuspended in LB, M9 + 5 mM acetate, M9 + 0.4% glucose without MgSO₄, and M9 + 4% glucose were incubated at 37 °C under aeration. After one hour, the bacteria were washed and resuspended in 1x PBS for flow cytometry (FACS) analysis. At least 500,000 events were recorded. The GFP signal was excited at 488 nm, and emission was detected at 525/40 nm (FITC channel) using a Beckman Coulter CytoFLEX S flow cytometer. The corresponding non-fluorescent wild type strain (8640) was used as a control for gating.

For the semiquantification of the induction of P*ssaG*-*gfp* (SPI-2) in macrophages, bacteria (SB214 = wild type; SB216 = *lepA* mutant) were grown to the stationary phase (OD_600_ = 2.0-2.5) and prepared as described for the invasion assays. Macrophages were infected at an MOI of 10. After 30 min of internalization, the wells were carefully washed three times with PBS and further incubated for 60 min in culture medium (IMDM + 10% FCS + 50 µg/ml gentamicin). Subsequently, the wells were again washed three times. After the third washing step, an aliquot was removed to measure the GFP signal in the supernatant. This aliquot was processed in parallel with the lysed macrophages. The macrophages were then lysed with Triton X-100 (0.1%). To maximize the release of the internalized bacteria, the macrophages were further incubated at room temperature for 10 min while shaking (1400 rpm). The cell debris was then removed by centrifugation (1000 x g for 1 min), and an aliquot was taken for further analysis. The samples were washed twice with PBS and then analysed by FACS as described for induction of P*prgH*-*gfp* above. At least 30,000 events were measured; however, no additional events were recorded because the number of measurable events in the supernatant was low. The corresponding non-fluorescent wild type strain (8640) was used as a control. The subpopulation of GFP-positive cells was gated to calculate the percentage of GFP-positive bacteria, and the relative frequency of SPI2-positive bacteria (mutant/WT) was calculated.

### RT-qPCR

Real-time PCR was used to quantify the expression of the giant adhesin *siiE*. Bacteria were grown in LB to the stationary phase (OD_600_ = 2.0–2.5), washed, and resuspended in DMEM supplemented with 10% FCS. Subsequently, the bacteria were incubated for one hour at 37 °C and 5% CO₂. After incubation, RNA was extracted using the Qiagen RNeasy Mini Kit according to the manufacturer’s protocol. The RNA was reverse-transcribed using random hexamer primers, 2 µl of 10 mM dNTPs (Invitrogen), 1 µl of 200 U/µl reverse transcriptase (Thermo Scientific), and 0.5 µl of 40 U/µl RiboLock RNase Inhibitor (Thermo Scientific). For real-time PCR, 5 ng of cDNA was used together with SYBR Green (Thermo Fisher Scientific) and primers for *siiE* (Table S3). Data were normalized to the housekeeping gene *trpA* [30, 31].

### Growth assays

The bacteria were streaked onto either LB plates or minimal medium plates containing 0.4% glucose to assess growth in minimal medium with the specified carbon sources. Plates were incubated overnight at 37 °C. The following day, the bacteria were resuspended in the corresponding medium (M9 + 1% peptone; M9 + 0.4% glucose; M9 + 0.2% maltose; M9 + 0.4% glycerol) and diluted to a concentration of 10^5^ cells/ml. Then, 1 ml of the suspension was transferred into a 48-well plate in triplicates. Growth was subsequently monitored using a BioTek Synergy HT plate reader and the lag time was calculated using BioTek Gen5 software.

### Lag Time

To determine the relative lag time of the bacteria, we used the BioTek Synergy HTX Multimode Reader and the accompanying Gen5 software. Cell division depends on the membrane potential [32]; when the membrane potential is low, cell division is delayed, resulting in an extended lag phase. To artificially prolong the lag time, we used the protonophore CCCP (carbonyl cyanide m-chlorophenyl hydrazone) to reduce the membrane potential. Bacterial cultures were prepared as described for the growth assays, and lag time was calculated using the Gen5 software.

### Motility Assay

Bacteria were incubated overnight at 37 °C on LB agar plates and subsequently resuspended in 1 ml LB. After appropriate dilution, 10⁶ bacteria were spotted onto soft agar (0.3%) and incubated for 6 h at 37 °C. Motility was determined by measuring the diameter of the colonies. An immotile *fljB fliC* mutant strain (9436) was used as a control.

### Microscopic images

To observe cell division in *Salmonella*, we utilized the plasmid pDR122, kindly provided by Dr. DeBoer from the Department of Molecular Biology and Microbiology at Case Western Reserve University. This plasmid encodes the *gfpmut2-t-minD-minE*, which is a MinD-GFP fusion protein [33]. The *Salmonella* strains carrying pDR122 (8640 pDR and SB6 pDR) were incubated in LB medium (100 µg/ml carbenicillin) until the OD_600_ reached approximately 0.5. Between 1 and 2 µl of the culture were then spotted onto soft LB agarose pads containing 50 ng/ml Isopropyl-β-D-thiogalactopyranoside (IPTG) and carbenicillin. The soft agarose pads were secured in place using silicone isolators (A/A) purchased from Electron Microscopy Sciences (13 mm diameter x 0.1 mm depth). The pads were subsequently covered with Zeiss coverslips (18 mm x 18 mm; 0.170 ± 0.005 mm thickness).

Confocal microscopy was performed at the Service Unit Microscopy of the Veterinary Centre for Resistance Research (TZR) with an inverted Leica Stellaris 8 FALCON microscope, equipped with a 405 nm laser, a white light laser (WLL, 440–790 nm), an acousto-optical beam splitter and Power HyD detectors. The microscopy was operated by LAS X version 4.5.0.25531.

Images were acquired with a HC PL APO 63x/1.30 oil immersion objective. Excitation of GFP was performed at 488 nm using the WLL set to 5.0% intensity, applying a unidirectional resonant scanning mode with 6 line accumulations at 8000 Hz scan speed. Fluorescence emission was collected between 498 and 750 nm using a HyD S detector in intensity mode (gain 10.1) and the pinhole set to two airy units. A brightfield image was acquired simultaneously using a Trans-PMT detector. A zoom of 5 was applied resulting in a pixel size of 72 nm and images consisting of 6 optical sections with a z-step size of 150 nm were acquired as time-series with an interval of 2.375 s. At least three randomly selected positions per strain were imaged consecutively at 37 °C for 5 min each. Post-processing of the GFP channel was performed using LAS X LIGHTNING with ‘Mounting Medium’ set to Water (RI = 1.33) and applying ‘Dynamic Signal Enhancement’ with the following settings: frames = 5, weight = 0.2 and periodicity = 1. Images were further processed linearly for brightness and contrast in ImageJ/FIJI [34] and are displayed as maximum intensity projections.

To analyze the intensity of GFP and the duration of the oscillation of the GFP-MinD fusion protein, we randomly selected at least three bacterial cells per observation across three independent experiments. We measured cell length using the analysis tools in ImageJ/Fiji. To determine the duration of one oscillation cycle from one cell pole to the other, we applied a z-axis profile and normalized it to the cell length. The GFP intensity was measured by analyzing the GFP signal over four oscillation cycles per cell to calculate the mean value.

### Infection of *Galleria mellonella*

Larvae were selected for the absence of melanization or other signs of illness. The larvae weighed approximately 300 mg during the infection assays. To determine pathogenicity, the bacteria were incubated as described for the invasion assays in LB until stationary phase. Subsequently, the bacteria were washed with PBS and diluted so that 10 µl contained approximately 10⁴ bacteria. This volume was injected into the hemolymph under the left proleg using a 30G insulin syringe. For each condition tested, 15 larvae were used, including a control group injected with 10 µl PBS. After injection, larvae were incubated at 37 °C, and at the indicated time points, viability was assessed by melanization and response to touch. Larvae were considered dead when no movement was observed. Data were presented as percent survival, and three independent experiments were conducted.

### Statistical analysis

All experiments were performed independently at least three times. Where indicated, an unpaired Student’s t-test was used to determine statistically significant differences. For the *Galleria mellonella* mortality assay, the log-rank (Mantel–Cox) test was used, with 15 larvae per experimental condition. A p-value of < 0.05 was considered statistically significant.

## Results

### Invasion deficiency of ∆*lepA* into host cells

**Fig. 1:**
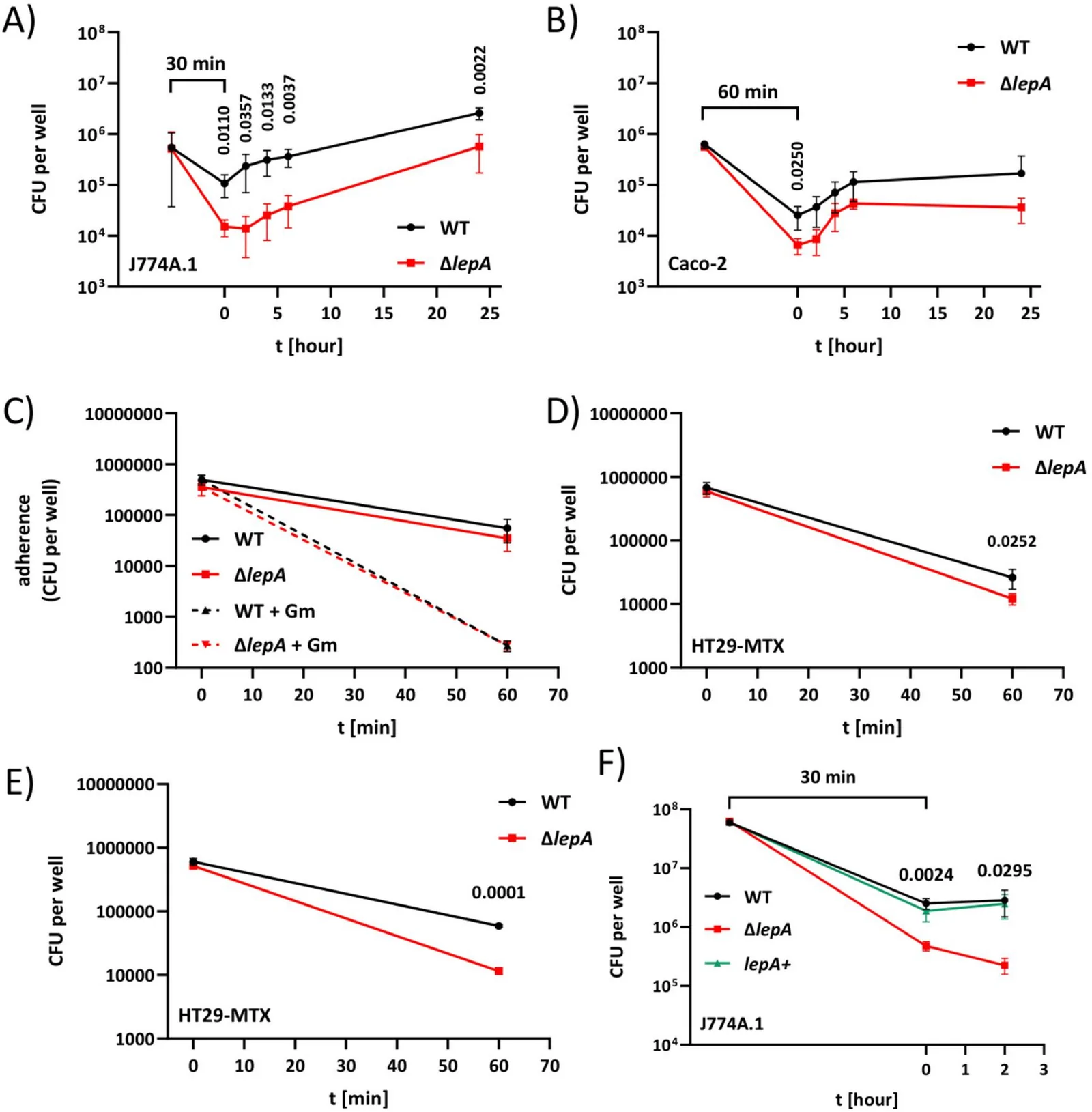
Invasion efficiency of *Salmonella* Typhimurium and the *lepA* mutant into host cells.** A** Wild type strain (8640) and the *lepA* mutant (SB6) were grown to stationary phase and used for invasion assays in J774.A1 (30 min incubation). A multiplicity of infection (MOI) of 10 was used, with experiments performed in duplicate for each indicated time point. **B** Invasion into Caco-2 cells (60 min incubation). The bracket indicates the invasion time, and t0 represents the number of invasive bacteria. An MOI of 10 was used, and each time point was conducted in duplicate. **C** Adherence assay with the wild type and the *lepA* mutant on Caco-2 cells. t0 indicates the initial bacterial CFU, and t60 indicates the number of bacteria attached to the Caco-2 cells after 60 min. As a control, 50 µg/ml gentamicin (Gm) was used to kill extracellular bacteria, allowing the distinction between intracellular and extracellular bacteria and ensuring that only attached bacteria were measured. An MOI of 10 was used in duplicate for each condition and strain. **D** Invasion into HT29-MTX cells lacking mucus production. Bacteria were incubated for 60 min to allow invasion into the epithelial cells. An MOI of 25 was used, and experiments were performed in duplicate. **E** Invasion after 21 days of incubation, allowing mucus layer formation on the HT29-MTX cell surface. t0 indicates the initial number of bacteria (MOI of 15 in duplicate), and t60 indicates the number of internalized bacteria after 60 min. **F** The wild type (8640), the *lepA* mutant (SB6), and the chromosomally complemented *lepA* strain (SB580) were grown to an OD_600_ of 1.0 and used to infect J774A.1 macrophages at a multiplicity of infection (MOI) of 10. For panel F), bacteria were grown to the post-exponential phase, whereas bacteria in panels A–E were grown to the stationary phase. In A), B), D), E) and F) after the indicated invasion time, cells were additionally treated with 50 µg/ml gentamicin for 30 min to kill extracellular bacteria before determining the CFU at time point t0. At least three independent experiments were performed in duplicate, and statistical significance was determined using an unpaired Student’s t-test (exact *p* values are indicated directly in the figure)

*Salmonella* is a facultative intracellular pathogen that preferentially replicates in macrophages to cause systemic infections [35]. Therefore, we conducted invasion assays using J774A.1 macrophages, a mouse macrophage-like cell line derived from a reticulum cell sarcoma, to test the ability of the Δ*lepA* mutant to invade and replicate within these cells. Macrophages were infected at a multiplicity of infection (MOI) of 10 in duplicate, with either the wild type or the Δ*lepA* strain. Following incubation, the macrophages were lysed, and invasive bacteria were plated on LB agar for CFU determination. Analysis of the results revealed that the wild type strain exhibited a sevenfold higher invasion efficiency compared to the Δ*lepA* mutant (*p* = 0.011; Fig. 1A). Furthermore, the initial intracellular growth of the Δ*lepA* strain was significantly impaired compared with that of the wild type, indicating that the mutant has impaired adaptation following invasion. The wild type was able to resume growth within the first two hours, whereas the mutant required at least four hours to initiate intracellular replication at a rate comparable to that of the wild type. To test whether this represents a general defect of the *lepA* mutant, we performed the same assay with Caco-2 cells, representing intestinal epithelial cells [36]. Similar to the macrophage-like cells, invasion of Caco-2 cells by the *lepA* mutant was reduced approximately threefold compared to the wild type (Fig. 1B). However, intracellular growth was unaffected and comparable to the wild type, indicating that the *lepA* mutant has specific difficulties coping with the hostile environment of immune cells (Fig. 1A and B) [13]. We also calculated the ratio between both strains after invasion into macrophages and epithelial cells to emphasize the difference (calculated as CFU WT / CFU mutant). After internalization into J774A.1 macrophages, the difference between the wild type and the *lepA* mutant increased from approximately sevenfold after invasion to 20-fold two hours post-invasion, whereas the ratio between both strains remained constant after invasion into Caco-2 cells (Supp Fig S1A and S1B). This finding further emphasizes that the hostile intracellular environment of immune cells is particularly detrimental to the *lepA* mutant during the initial phase of infection.

The decreased invasion also cannot be explained by defective adhesion to host cells, as no significant differences were observed in surface binding to Caco-2 cells (Fig. 1C). As a gastrointestinal pathogen, *Salmonella* must cross the mucus layer covering the intestinal epithelium, which consists of approximately 95% water and 5% highly glycosylated mucin glycoproteins, as well as salts, lipids, and protective proteins [37–39]. Therefore, we conducted invasion assays using HT29-MTX cells (without determining intracellular growth, as HT29-MTX cells are comparable to Caco-2), which differentiate into mucus-producing, goblet cell–like cells [40]. In the absence of mucus, invasion of the wild type was slightly but significantly higher (approximately two-fold) than that of the mutant after 60 min (Fig. 1D). After prolonged incubation of HT29-MTX cells to allow mucus formation, invasion of the *lepA* mutant was again significantly reduced, by approximately fivefold, compared to the wild type (Fig. 1E). Interestingly, wild type invasion increased in the presence of mucus, indicating that *Salmonella* is well adapted to this environment (Gagnon et al., 2013, [41]).

We also tested invasion into host cells after incubation to the post-exponential phase (OD_600_ = 1.0), corresponding to the onset of SPI-1 invasion gene induction, to determine whether the impaired invasion into macrophages and epithelial cells is growth-phase dependent. After infection of macrophages, invasion was reduced following deletion of *lepA*, as was initial intracellular replication (Fig. 1F). This impaired phenotype could be restored by chromosomal complementation, indicating that *lepA* is responsible for the reduced invasion and replication (Fig. 1F). Invasion assays with Caco-2 cells also showed reduced invasion (two-fold); however, the difference was not significant (Supp Fig S2).

In summary, deletion of *lepA* results in reduced invasion across various host cell types and impairs initial intracellular growth in macrophages.

### Reduced activation of virulence factors in the Δ*lepA* mutant

**Fig. 2:**
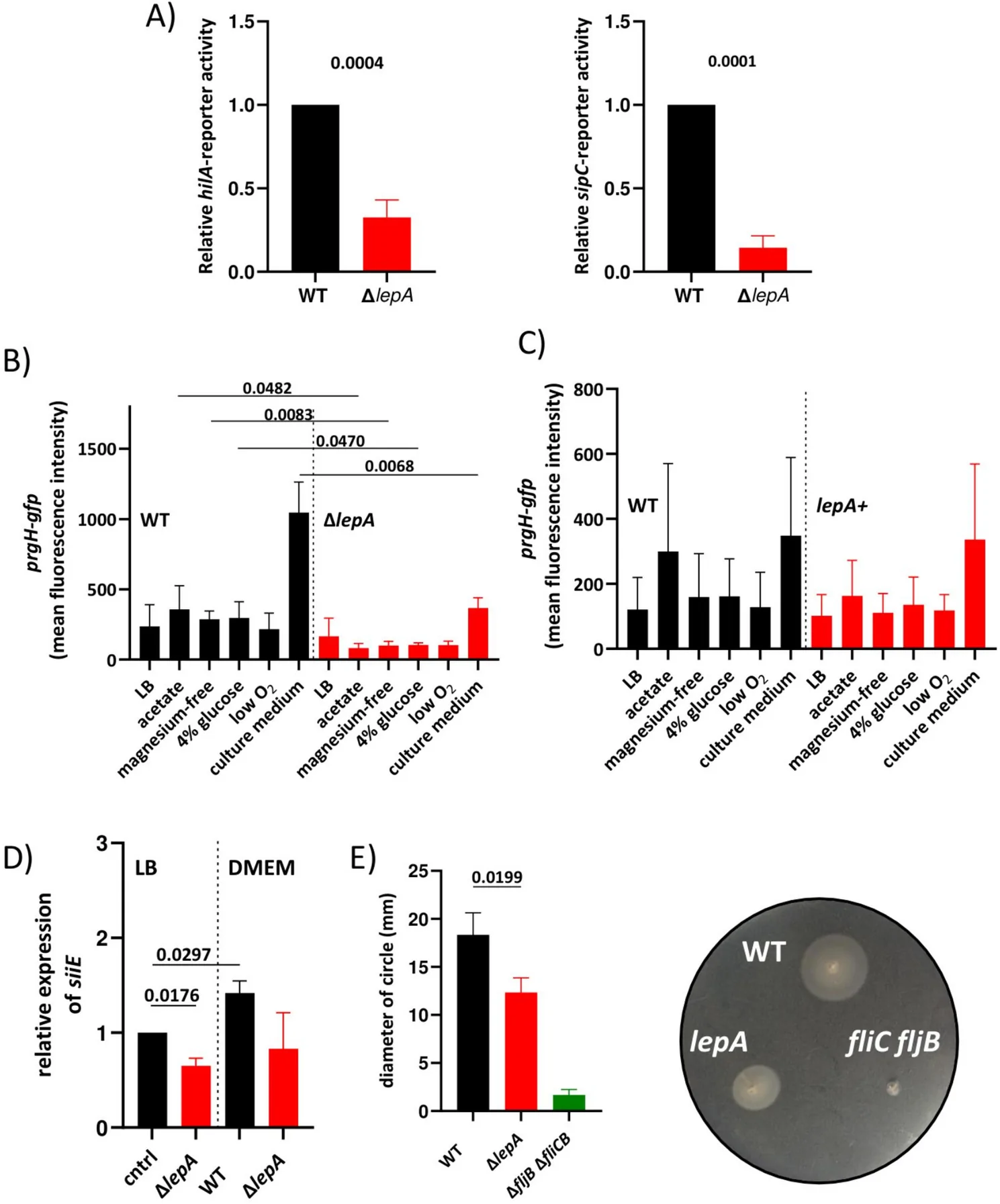
Assessment of SPI-1 expression and additional virulence factors in the*lepA*mutant.** A** LacZ assays using *hilA*::*lacZ* and *sipC*::*lacZ* reporter strains. The wild type (SB473 = *hilA*::*lacZ* and SB478 = *sipC*::*lacZ*) and the *lepA* mutant (SB474 = *hilA*::*lacZ* and SB479 = *sipC*::*lacZ*) were grown in LB medium to an optical density of 2–2.5 at 600 nm before measuring β-galactosidase activity. The calculated Miller Units were normalized to the respective wild type. **B** For subpopulation-level analysis, a P*prgH*-*gfp* reporter strain was used. Bacteria (wild type = SB210; Δ*lepA* = SB212) were grown in LB as described in A), centrifuged, and resuspended in one of the following media: M9 supplemented with 5 mM acetate (acetate), M9 with 0.4% glucose but without magnesium (Mg-free), M9 with 4% glucose (OS, osmotic stress), M9 with 0.4% glucose under microaerophilic conditions (low O₂), or DMEM (cell culture medium). The GFP signal was then quantified by flow cytometry (FACS). A representative histogram from the FACS analysis is shown in Supp Fig S3A. **C** Measurement of SPI-1 induction in the wild type (8640) and the chromosomally complemented *lepA*+ strain (SB580) following growth to the stationary phase (OD_600_ = 2.0-2.5) and subsequent incubation under the indicated conditions for 1 h. **D** RT-qPCR of the wild type and the *lepA* mutant after incubation in LB and DMEM to determine the induction of the giant adhesin *siiE*. The results were normalized to the wild type (“cntrl”) incubated in LB. **E** Motility assay on LB soft agar (0.3%). As a control, the non-motile *fliC fljB* mutant (9436) was used. To the right is a representative image of the motility assay. At least three independent experiments were performed, and statistical significance was determined using an unpaired Student’s t-test (exact *p* values are indicated directly in the figure)

SPI-1 is a major virulence factor that plays a critical role in causing systemic infections following oral administration [8]. Therefore, to further investigate the impaired invasion efficiency, we performed lacZ assays in which the promoters of *hilA*, the master regulator of SPI-1, and *sipC*, a component of the T3SS, control β-galactosidase expression [8, 42]. The bacteria were incubated as described for the invasion assays in LB until they reached the appropriate cell density, followed by measurement of β-galactosidase activity. For both *hilA* and *sipC*, expression was approximately 70–80% lower in the Δ*lepA* mutant than in the wild type, indicating that SPI-associated gene expression is already reduced in the Δ*lepA* mutant prior to the invasion assay (Fig. 2A). To further investigate this difference, we also employed a chromosomal P*prgH*-*gfp* reporter strain, in which *gfp* expression is driven by the promoter of the T3SS-1 *prg* operon [27]. This approach allowed us to assess SPI-1 induction under various stress conditions. Again, bacteria were grown to the stationary phase, washed with PBS, and resuspended in one of the following media: M9 + 0.4% glucose, M9 + 4% glucose, M9 + 0.4% glucose without MgSO₄, M9 + 5 mM acetate, DMEM + 10% FCS, or LB. These conditions were chosen to analyze the effect of *lepA* deletion on SPI-1 induction under known SPI-1–modulating environments: short-chain fatty acids (acetate) [43], low oxygen (microaerophilic) [8], magnesium limitation (Mg²⁺-free) (Altier, [44]), osmotic stress (4% glucose) [45], host-like conditions (DMEM), and nutrient-rich medium (LB). After one hour of incubation under the respective conditions, bacteria were prepared for FACS analysis. Under all tested conditions, SPI-1 induction was higher in the wild type (Fig. 2B). In particular, under stress conditions (acetate, osmotic stress, low magnesium, and low O₂), the proportion of the population exhibiting SPI-1 induction was reduced or unchanged in the *lepA* mutant compared to that observed in nutrient-rich LB medium (Supp Fig S3A). In the wild type, SPI-1 expression was upregulated under stress conditions compared to LB, ranging from 1.2- to 3.65-fold. The only exception was incubation in M9 + 0.4% glucose under microaerophilic conditions, which led to a slight downregulation (0.9-fold). In contrast, in the Δ*lepA* mutant, SPI-1 was downregulated under all stress conditions and showed only a modest 1.78-fold increase in DMEM compared to LB (Fig. 2B). We also retested our chromosomally complemented strain and did not detect a statistically significant difference in P*prgH*-*gfp* expression compared with the wild type, which is consistent with the invasion results observed in macrophages (Fig. 2C). We also tested the induction of our P*prgH*-*gfp* reporter after incubation to the post-exponential phase, using the same conditions as described above (M9 + 0.4% glucose, M9 + 4% glucose, M9 + 0.4% glucose without MgSO₄, M9 + 5 mM acetate, DMEM + 10% FCS, and LB). As before, induction was weaker in the *lepA* mutant under the various conditions, including the acetate condition (Supp Fig S3B). Overall, P*prgH*-*gfp* expression was higher in the stationary phase than in the post-exponential phase, indicating that SPI-1 expression is modulated according to the bacterial growth phase. These findings indicate that, prior to invasion, SPI-1-associated gene expression is substantially higher in the wild type than in the *lepA* mutant, suggesting that the absence of *lepA* affects SPI-1 expression and may consequently impair proper T3SS-1 function. This suggests that *lepA* plays an important role under stress conditions and may contribute to the regulation of SPI-1 expression, potentially at the translational level.

Because replication of the *lepA* mutant was disturbed after invasion into macrophages, we also semiquantified SPI-2 induction using the reporter strain P*ssaG*-*gfp* [27]. For this, we incubated the bacteria to the stationary phase and infected macrophages as described before. To minimize the effect of extracellular bacteria, we washed the macrophages three times with PBS 30 min after addition of the bacteria and again after an additional incubation time of 60 min with 50 µg/ml gentamicin. After the last washing step, an aliquot was removed and processed in parallel with the lysed macrophages. After removal of cell debris by centrifugation and washing with PBS, the bacteria were analysed by flow cytometry. The non-fluorescent wild type strain (8640) as well as both GFP-fluorescent strains (wild type and mutant) were analysed, and the subpopulation with a positive GFP signal was determined. Although extracellular bacteria may still have contributed to the signal, the results strongly indicated that the subpopulation with SPI-2 induction after internalization into macrophages is approximately twofold higher in the wild type compared to the *lepA* mutant (Supp Fig S4A and S4B). However, more refined experimental approaches will be required to unequivocally confirm these observations and to precisely quantify SPI-2 induction specifically within intracellular bacteria.

In addition to SPI-1 and SPI-2, further virulence factors are important for the pathogenicity of *Salmonella*. The giant adhesin SiiE plays a crucial role in the invasion of HT29-MTX cells by binding to MUC1, a transmembrane mucin on the host cell surface, thereby positioning the bacteria for the injection of SPI-1 effector proteins [41]. Therefore, we also examined the transcriptional activation of *siiE* in LB and after one hour of incubation in DMEM. The results indicate a significant upregulation of *siiE* after one hour of incubation in DMEM for the wild type compared to LB (Fig. 2D), whereas no upregulation was observed for the *lepA* mutant, thus partially explaining its impaired invasion of HT29-MTX cells (Fig. 1D and E).

Another important aspect of *Salmonella’s* pathogenic efficiency is motility, particularly when encountering mucus in the intestines. Through flagellar propulsion, *Salmonella* actively searches for breaches in the mucus layer to cross this barrier and invade the underlying epithelial cells [37]. To assess the intrinsic motility capacity of the strains under standardized baseline conditions, bacteria were incubated on LB-based soft agar (0.3%) for six hours at 37 °C, and the diameter of the motility halo was measured. To verify the reliability of the assay, a non-motile *fliC fljB* mutant was tested. The assays revealed that the wild type moves at a rate of 1130 μm/h, whereas the *lepA* mutant moves at approximately 1000 μm/h. Thus, the flagella-driven motility of the *lepA* mutant was significantly reduced (Fig. 2E), demonstrating that deletion of *lepA* not only interferes with SPI-1/SPI-2 induction but also affects additional important virulence factors, such as activation of the adhesin *siiE* and bacterial motility.

### Infection of *Galleria mellonella*

**Fig. 3:**
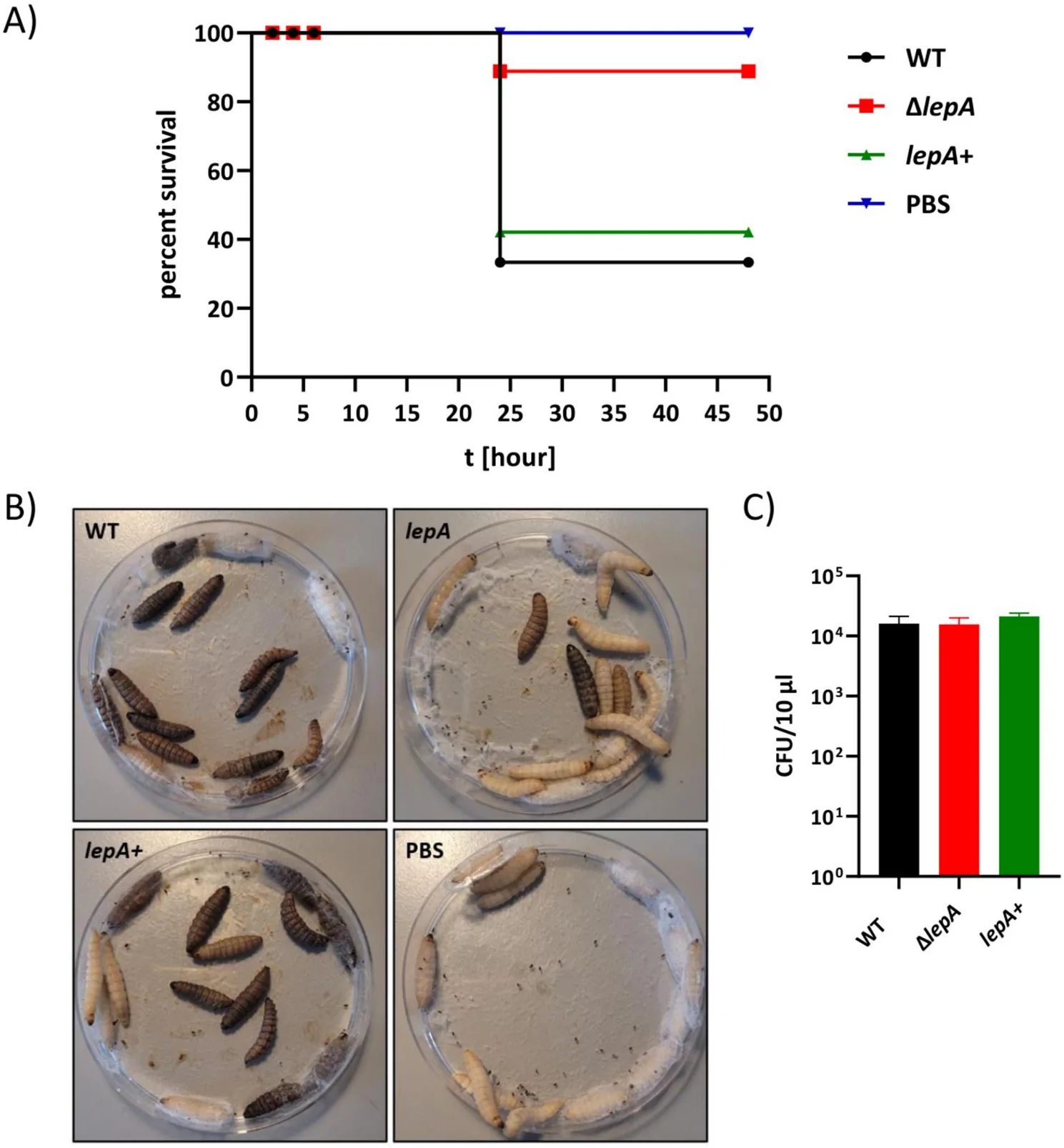
Infection of *Galleria mellonella* with *Salmonella* Typhimurium.** A** The bacteria (wild type = 8640; *lepA* = SB6; *lepA* + = SB580) were grown to an optical density at 600 nm of 2.0–2.5 before injection into the larvae. Mortality was assessed at the indicated time points. Significant differences were determined using the Log-rank (Mantel–Cox) test and are as follows: wild type vs. *lepA*, *p* < 0.0001; wild type vs. *lepA*+, *p* = 0.5875; *lepA* + vs. *lepA*, *p* = 0.0008. PBS was used as a control to ensure that the injection and handling were not lethal. In addition, a chromosomally complemented *lepA* strain (*lepA*+) was included. Three independent experiments, each with 15 larvae per strain, were conducted, and one representative experiment is shown. **B** Dead larvae appear black, are immobile, and do not respond to external stimuli. **C** The larvae were injected with approximately 10⁴ bacteria per 10 µl

To test the *lepA* mutant under more physiologically relevant conditions, we performed infection assays using *Galleria mellonella* larvae. These larvae possess an innate immune system similar to that of mammals, including phagocytic hemocytes that produce lysozymes for bacterial degradation, generate reactive oxygen species, and secrete antimicrobial peptides to eliminate pathogens [46]. This infection model has also been previously established for *Salmonella* [47]. The bacteria were grown to stationary phase in LB, washed with PBS, and diluted accordingly. Subsequently, 10 µl containing approximately 10⁴ bacteria were injected into 15 larvae per condition. After injection, the larvae were incubated at 37 °C, and mortality was assessed at the time points indicated in Fig. 3A. These infection assays revealed that mortality was significantly higher in larvae infected with the wild type strain. Approximately 70% of the wild type-infected larvae died within 24 h, whereas only 10–20% of the larvae infected with the *lepA* mutant died. We also tested a chromosomally complemented *lepA* strain (*lepA*+), which showed pathogenicity comparable to the wild type, underscoring the importance of LepA for *Salmonella* virulence. Larvae were considered dead when they showed no response to external stimuli, with melanization serving as an additional visible indicator of disease progression (Fig. 3B). To ensure that the correct number of bacteria was injected, CFU counts were determined prior to infection (Fig. 3C).

### Growth impairment of *lepA* mutants

We were also interested in whether *lepA* deletion affects other aspects of *Salmonella* biology beyond virulence. Therefore, we performed growth assays using various carbon sources and found that, under all tested conditions, growth of the *lepA* mutant was slightly impaired, indicating that LepA has a general impact on bacterial metabolism (Supp Fig S5). We also analyzed the lag phase to evaluate growth resumption of the *lepA* mutant, which was likewise affected when incubated in LB (Supp Fig S6). Upon addition of CCCP, a protonophore that reduces the membrane potential, the lag phase was prolonged more severely in the *lepA* mutant than in the wild type. These findings suggest that deletion of *lepA* may affect bacterial adaptation to perturbations in membrane energetics or proton motive force (PMF) homeostasis. These results are also consistent with disturbed initial respiratory activity in *S*. Typhimurium [17]. We also investigated whether the cell division apparatus was affected by *lepA* deletion using an IPTG-inducible MinD-GFP fusion protein (P*lac*::*gfpmut2*-*t*-*minD-minE*), as the elongated lag time suggested a decreased membrane potential, which plays a significant role in cell division and the proper localization of cell division proteins [32]. However, artificial induction with IPTG did not result in any significant differences (Supp Fig S7 and Movie S1). We measured the oscillation time as well as the intensity of the GFP signal. The average GFP-MinD oscillation cycle lasted 17 s at 37 °C and 27 s at 28 °C in both strains, respectively. These results indicate that *lepA* deletion does not significantly affect MinD-mediated cell division under the conditions tested. In the future, it would be interesting to investigate more elaborate approaches, such as transcriptomic or proteomic analyses, to determine the effect of LepA on bacterial metabolism and to further underscore the importance of LepA and explain the high conservation of this protein.

## Discussion

The food-borne pathogen *Salmonella* Typhimurium has a complex regulation of its virulence genes to ensure induction at the appropriate time. After infecting humans and animals, *Salmonella* encounters different physiological changes regarding oxygen and nutrient availability, as well as the presence of bile acids, which stimulates the activation and expression of SPI-1 genes [8]. This enables *Salmonella* to invade epithelial cells and macrophages, replicate within them, and subsequently disseminate systemically via the lymphatic system [48]. These virulence genes consume considerable resources in a nutrient-limited environment, where *Salmonella* must compete with the natural gut microbiota. Therefore, efficient activation and formation of the T3SS (Type III Secretion System) are likely crucial for successful invasion, replication, and dissemination. The T3SS is an intricate enzyme complex consisting of several proteins that span both the inner and outer membranes of *Salmonella* [8]. Considering the reported involvement of LepA in translation and its potential role in translational accuracy, deletion of *lepA* may affect the efficiency or fidelity of protein synthesis. Such effects could become particularly relevant for complex regulatory networks such as SPI-1, where small changes in the abundance or functional activity of key regulatory proteins could propagate through the regulatory cascade and weaken SPI-1 gene expression. This may contribute to the reduced GFP induction observed in the Δ*lepA* P*prgH*-*gfp* reporter strain under various conditions (Fig. 2B). Impaired translation or protein function could consequently affect T3SS expression and assembly, potentially contributing to the reduced invasion phenotype. However, this possibility remains speculative and requires further investigation at the protein and functional levels.

For macrophages, a large proportion of bacteria is actively taken up by phagocytosis, yet SPI-1 still plays a crucial role in initiating their invasion [9, 49]. Therefore, impaired SPI-1 induction in the l*epA* mutant likely contributes to its reduced invasion efficiency into J774A.1 macrophages. However, the mutant also shows a significant delay in growth resumption after internalization, suggesting difficulties in establishing a replicative niche (Fig. 1A and F). This may indicate that a fraction of the Δ*lepA* bacteria is killed after uptake, potentially due to impaired prevention of *Salmonella*-containing vacuole fusion with lysosomes, resulting in bacterial degradation. This is supported by the weaker induction of SPI-2 in the *lepA* mutant following internalization (Supp Fig S4). Importantly, not all bacteria need to induce SPI-2 to prevent lysosomal fusion, as SPI-2-expressing bacteria can also protect dormant or non-SPI-2-induced cells within the same population (Pospisilova et al., 2025). However, deletion of *lepA* appears to reduce the proportion of SPI-2-positive cells, thereby diminishing the overall protective effect against phagolysosome formation and subsequent enzymatic degradation.

Invasion into Caco-2 cells was also impaired after deletion of *lepA*, while intracellular growth remained unaffected (Fig. 1B). This suggests that the Δ*lepA* strain has difficulties circumventing the aggressive intracellular environment of immune cells. Furthermore, it has been reported that a significant portion of SPI-1 mutants is still able to invade Caco-2 cells, indicating the existence of a second, SPI-1-independent invasion mechanism [50]. This implies that LepA influences not only the induction of SPI-1 but also other virulence-related factors, contributing to the reduced invasion into Caco-2 cells. It also emphasizes the redundancy of *Salmonella’s* invasion process, which does not rely on a single mechanism. In addition to SPI-1, several other factors play crucial roles in *Salmonella* pathogenesis, including cell surface structures such as fimbriae/adhesins (Baumler et al., 1996), lipopolysaccharides [47], the metabolic state of the bacteria (Kim et al., 2013), and active uptake by host cells, for example via pinocytosis (Garcia-del Portillo and Finlay, 1994). In addition, we tested invasion after growth to the post-exponential phase (OD₆₀₀ = 1.0). Under these conditions, the difference between the wild type and the mutant was less pronounced, with approximately twofold higher invasion by the wild type that did not reach statistical significance (Supp Fig S2). However, SPI-1 induction, measured using the SPI-1-*gfp* reporter, was significantly increased in the wild type (Supp Fig S3B). This suggests that LepA may contribute to Caco-2 cell invasion through mechanisms at least partially independent of SPI-1 expression. Thus, the combination of inefficient SPI-1 induction and defects in additional virulence factors may contribute to impaired invasion. Similar defects were observed in HT29-MTX cells, potentially due to both reduced SPI-1 activation and decreased expression of the giant adhesin SiiE, which interacts with MUC1 to position the T3SS for effector injection [41]. Furthermore, deletion of *lepA* also impaired motility, which is an important virulence mechanism required to cross the intestinal mucus barrier and reach the underlying epithelial cells. In *Salmonella*, flagellar gene expression and SPI-1-mediated virulence are generally considered to be inversely regulated [51]. However, the reduced motility of the *lepA* mutant cannot be attributed to a compensatory upregulation of the SPI-1 regulon, as SPI-1-associated gene expression was concurrently decreased in the mutant compared to the wild type. These data indicate that deletion of *lepA* leads to a synchronous down-regulation of both bacterial motility and SPI-1/SPI-2 induction. Thus, multiple factors are affected that reduce the pathogenicity of Δ*lepA* and explain the decreased invasion.

In addition to these virulence-associated phenotypes, deletion of *lepA* was also associated with subtle changes in bacterial growth dynamics. The lag phase, which is the time required to transition from a non-growing state to exponential growth, is approximately 45 min longer in LB after deletion of *lepA* (Supp Fig S5A). Similar effects were observed in minimal medium supplemented with either glucose, peptone, maltose, or glycerol (Supp Fig S5C). At 22 °C in LB, the lag phase of the *lepA* mutant is extended by 120 min, consistent with previous reports indicating that LepA plays a crucial role under stress conditions, such as high ionic stress or low temperature (Supp Fig S5B) [22]. These growth results suggest that LepA contributes to efficient adaptation to new environmental conditions, potentially by supporting translational responses required for optimal growth. However, the growth defect is unlikely to fully account for the reduced invasion efficiency of the *lepA* mutant. In particular, the difference in invasion between the wild type and the *lepA* mutant was further increased in the presence of mucus, suggesting that additional mechanisms contribute to the impaired invasion phenotype. The reduced induction of SPI-1, impaired motility, and decreased *siiE* expression may therefore play a more important role in the reduced invasion efficiency of the *lepA* mutant.

To further investigate the underlying cause of the prolonged lag phase, we used a plasmid encoding a GFP-MinD fusion protein activated by the addition of IPTG [33]. The *min* operon is essential for cell division, oscillating from one cell pole to the other to ensure proper FtsZ ring formation at the cell’s center during division [33]. The efficiency of the MinD/MinE system depends on the membrane potential [32]. However, artificial induction with IPTG did not result in any significant differences (Supp Fig S7 and Movie S1). The exact mechanism by which LepA reduces lag time and facilitates adaptation to new environmental conditions remains elusive.

The impaired pathogenicity of the *lepA* mutant was further confirmed in *Galleria mellonella* infection assays (Fig. 3). While approximately 25% of larvae infected with the wild type survived, survival increased to around 80% upon infection with the *lepA* mutant. Notably, SPI-1 and SPI-2 are dispensable for *G*. *mellonella* infection, whereas the two-component system PhoPQ is crucial for virulence, likely by modifying lipopolysaccharide and thereby increasing resistance to antimicrobial peptides [47]. This, in turn, raises the possibility that the *lepA* mutant may exhibit alterations in PhoPQ activation or lipopolysaccharide remodeling. Interestingly, *phoPQ* mutants are also more susceptible to macrophage-mediated killing and are avirulent in BALB/c mice [52, 53]. Consequently, the *lepA* mutant may potentially also exhibit reduced virulence in mouse infection experiments. These results indicate that LepA is not a classical virulence-associated protein but contributes to bacterial pathogenicity.

The *lepA* mutant shows impaired adaptation to changing environmental conditions, reduced induction of SPI-1 and *siiE*, decreased motility, and reduced pathogenicity in *G*. *mellonella*. Given the high conservation of LepA across bacteria and eukaryotic cells [24] these findings suggest that LepA may broadly contribute to metabolic adaptation and virulence.

## Supplementary Information

Below is the link to the electronic supplementary material.

Supplementary Material 1

Supplementary Material 2

## Data Availability

All data generated or analysed during this study have been included in the supplementary data (RawData).
